# Noninvasive Quantitative Compression Ultrasound Central Venous Pressure: A Clinical Pilot Study

**DOI:** 10.34133/bmef.0115

**Published:** 2025-03-19

**Authors:** Alex T. Jaffe, Roger Pallarès-López, Jeffrey K. Raines, Aaron D. Aguirre, Brian W. Anthony

**Affiliations:** ^1^ Department of Electrical Engineering and Computer Science, Massachusetts Institute of Technology, Cambridge, MA, USA.; ^2^ Department of Mechanical Engineering, Massachusetts Institute of Technology, Cambridge, MA, USA.; ^3^ Cardiology Division, Massachusetts General Hospital, Harvard Medical School, Boston, MA, USA.

## Abstract

**Objective:** This is an initial study to validate central venous pressure (CVP) measurements derived from quantitative compression ultrasound (QCU). **Impact Statement:** This study is the first gold standard invasive validation of CVP estimation from QCU. **Introduction:** QCU finds the collapse force—the force required for complete occlusion—of the short axis of the internal jugular vein (IJV) to estimate CVP. **Methods:** We captured QCU data as well as the noninvasive clinical standard jugular venous pulsation height (JVP) on cardiac intensive care unit (CICU) patients at Massachusetts General Hospital (MGH). We compared these data to ground truth invasive CVP data from the MGH CICU. **Results:** Using linear regression, we correlated invasive CVP with collapse force (*r*^2^: 0.82, error: 1.08 mmHg) and with JVP (*r*^2^: 0.45, error: 1.39 mmHg). To directly compare our method to JVP, we measured the percentage of patients whose uncertainty estimates for QCU methods and for JVP overlapped with their invasive CVP counterparts. We found that the CVP overlap accuracy of collapse force (77.8%) and of collapse force and hydrostatic offset (88.9%) are higher than that of JVP (12.5%). Finally, we input QCU image segmentation data of the short-axis cross-sections of the IJV and carotid artery into an inverse finite element model to predict the invasive CVP waveform. **Conclusion:** These results validate the noninvasive technique for estimating CVP, namely, QCU, indicating that it may provide a desirable, middle-ground alternative to invasive catheterization and to visual inspection of the JVP.

## Introduction

The measurement of central venous pressure (CVP) plays an important role in the comprehensive assessment and management of patients with signs and symptoms of heart failure, sepsis, and other forms of circulatory compromise, and as part of the monitoring protocol during many surgical interventions. CVP provides valuable insights for intravascular volume status, cardiac function, and overall hemodynamic status [1,2]. Patients in the intensive care unit (ICU) frequently have compromised cardiac function, fluid imbalances, and multi-system organ dysfunction [3–5]. Monitoring CVP in these cases becomes crucial in guiding therapeutic interventions, such as titrating vasopressor medications or administering diuretics [6–8]. Moreover, CVP measurements can aid in detecting and managing of conditions like sepsis, cardiogenic shock, and pulmonary edema [1,9].

The direct measurement of pressure in the superior vena cava or right atrium through catheterization serves as the gold standard for assessing CVP [10,11]. However, in relatively stable cases, noninvasive methods are utilized [10,12]. The 2 standard noninvasive techniques used for venous pressure assessment are direct visual measurement of jugular venous pulsation height (JVP) and measurement of inferior vena cava diameter using ultrasound [12,13]. While these methods are grounded in physiological principles, they have limitations in providing a reliable noninvasive standard for guiding treatment decisions. Moreover, JVP is somewhat dependent on the patient’s inclination, a variable that is difficult to alter in cardiac intensive care unit (CICU) settings, and has been shown to have substantial inter-operator variability [12,14]. As a result, considerable efforts have been made to enhance the assessment of venous pressure, with particular focus on advancements in medical ultrasound [15–19].

Quantitative compression ultrasound (QCU), also known as force-coupled ultrasound, is a novel technique that combines ultrasound imaging with simultaneous measurement of the force applied by the probe’s imaging surface on the skin over the target area [20,21]. This technique has been used for estimating blood pressure in the carotid artery [22–24]. Due to the lower intravascular pressure and stiffness properties of the internal jugular vein (IJV) compared to the carotid artery, a relatively low force can be used to completely occlude a short-axis cross-section of the IJV. In fact, this technique is routinely used clinically during vascular access procedures to correctly identify the veins versus the arteries. We call the compression force required to fully occlude the IJV the collapse force (CF) [24], and we have previously demonstrated that measurement of the CF with a force-coupled single-element ultrasound transducer can be used to quantify relative variations in jugular venous pressure [25].

We subsequently developed a method to estimate the mean CVP in healthy individuals using QCU [26]. Two main limitations constrained the prior studies. First, the visual estimation of JVP was used as the main standard of comparison, without measurements from invasive monitoring. Second, only healthy volunteers were recruited, which provided a limited range of CVPs (1.5 to 5 mmHg). CVP from hospitalized patients with cardiovascular disease may be much higher depending on intravascular volume status.

In this work, we present a clinical pilot study where we compare QCU measurements in the IJV with invasive CVP measured with standard-of-care central venous catheter placement. We collected data from 11 patients in the Massachusetts General Hospital (MGH) CICU and analyzed the relationship between variations in IJV CF measurements and invasive CVP measurements. We determine the accuracy and uncertainty metrics of our noninvasive measurements using the current invasive standard for assessing CVP. We also construct an inverse finite element model, which relies on QCU of the carotid artery and IJV to yield an estimate of the continuous CVP waveform and compare it to the invasive CVP waveform. Our findings suggest a strong relationship between QCU-based CF of the IJV and CVP, paving the way for future larger-scale studies to further validate and generalize the findings presented here.

## Results

Subjects were enrolled from those hospitalized in the MGH CICU with an indwelling central venous catheter as part of standard of care. Patient demographics are presented in Table 1.

**Table 1.** Demographics table. Each patient + mean (SD) of age, height, weight, and % of males/females.

Subject	Age (years)	Height (cm)	Weight (kg)	Sex
1	43	165	63.9	Male
2	48	191	118.7	Male
3	55	178	84.5	Male
4	53	173	98.1	Male
5	78	175	77.7	Male
6	81	166	86.3	Male
7	63	163	89.6	Female
8	78	180	78.2	Male
9	78	170	75.9	Male
10	53	170	93.1	Female
11	70	168	80.1	Male
Summaries	64 (13)	173 (8)	86.0 (14.3)	18% female

**Fig. 1.**
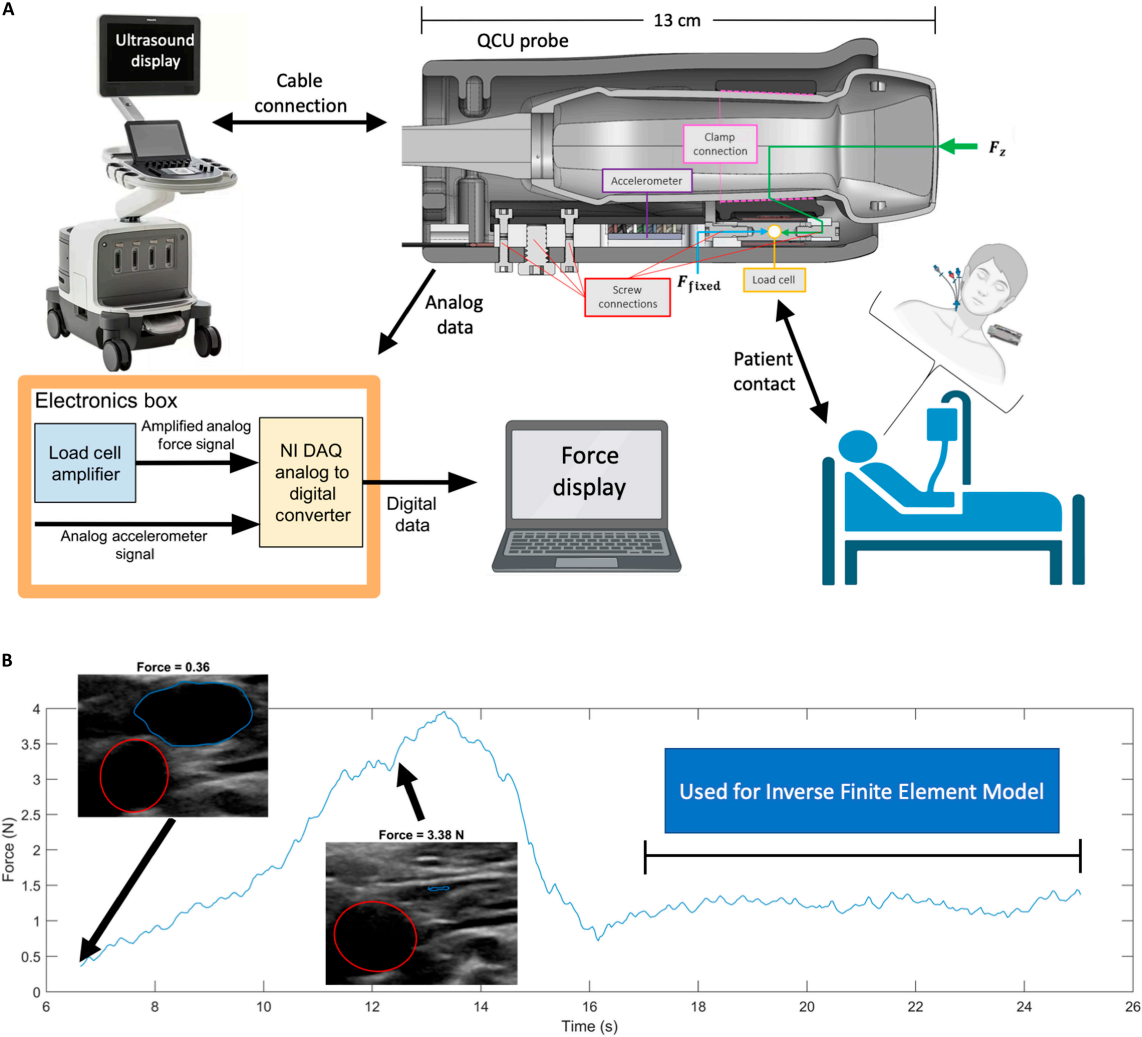
Data capture overview. (A) Quantitative compression ultrasound (QCU) imaging data collection flowchart: Force is measured, and ultrasound images are obtained from the QCU probe on the top right—shown as a computer-aided design (CAD) model cross-section of the Philips XL14-3 ultrasound probe with a force-coupling casing around it. The length from imaging surface to cable is provided as 13 cm. An illustration shows the placement of the ultrasound probe on the patient contralateral to catheterization. The force is translated from the green arrow at the imaging surface of the ultrasound probe to the load cell that measures force. (B) A force time series is shown to illustrate the data collection method. First, force is ramped from low to high until the IJV completely occludes, contributing to the CVP scalar estimate. Segmented QCU images of the carotid artery (red) and IJV (blue) are shown at low force and CF. Second, the force is held constant with the IJV in a lightly compressed state to capture carotid and IJV data to input into an inverse model that estimates the CVP waveform. Parts of this figure were created with BioRender.

The dataset contains noninvasive QCU measurements as well as invasive CVP waveforms and measurements. QCU data include short-axis, cross-sectional ultrasound images of the contralateral IJV from the one with the indwelling central venous catheter. Measurements of the direct force applied to the surface of the skin are taken simultaneously with ultrasound images. The QCU protocol allows direct measurement of the force necessary to fully collapse the target structure—the IJV short-axis cross-section. Figure 1A is an illustration of the ultrasound probe, which has been modified to provide quantitative measurement of probe force (Newtons) applied to the surface of the skin, and of how the measurements are processed. The force data derived from the probe (Phillips XL14-3 xMATRIX) are logged and analyzed by the Electronics Box and displayed via a laptop computer.

The procedure for acquiring these ultrasound data is further illustrated in Fig. 1B. The sonographer identified the short axis of the IJV and increased the probe compression until observing the complete occlusion of the IJV short-axis cross-section. This maneuver is very similar in force to the compression maneuver used by clinicians when identifying the IJV for placement of an intravenous catheter. To complete the process, the sonographer held force constant with partial IJV compression to collect data necessary for the inverse finite element model for CVP waveform estimation. A video of the QCU acquisition protocol is captured with annotations for further analysis. An example of such is provided in Movie S1.

In all cases, a central venous catheter was in place in the contralateral IJV as part of standard of care. The imaging protocol, in advance of compression, includes evaluation of the catheter location and height of the pressure transducer relative to the patient to ensure appropriate calibration. Figure S1 shows examples of subject CICU measurement data for catheter-determined right atrial pressure and a single electrocardiogram (ECG) lead. In 2 patients, partial venous thromboses were identified (subjects 6 and 8). In these cases, compression of the IJV was not performed and the external jugular vein (EJV) was compressed instead. All QCU measurements were made by staff physicians trained in vascular ultrasound.

The data we obtained are given in Table 2. QCU is first used to fit CF measurements to invasive CVP measurements. Then, JVP is used to estimate invasive CVP. Finally, a trained model from a previous study is used with QCU measurements from this study to estimate invasive CVP.

**Table 2.** Summary of CVP measurements and estimates made during the study for all subjects. Dashes symbolize JVP measurements not attempted. X’s symbolize JVP measurements attempted but unable to measure. The “Vein” column lists the vein measured for each subject. LIJ, left IJV; RIJ, right IJV; LEJ, left EJV; REJ, right EJV; #, subject number; Avg., average; CF, collapse force; HO, hydrostatic offset; Pred., predicted.

#	Avg. invasive CVP (mmHg)	Vein	CF (N)	HO (cmH_2_O)	CF Pred. CVP (mmHg)	Multi-feature Pred. CVP (mmHg)	JVP (mmHg)	Trained model CVP (mmHg)
1	8.5	LIJ	9.73	2.76	10.14	9.94	-	6.13
2	5.0	LIJ	3.66	5.00	3.78	4.03	3.68	5.25
3	4.5	LIJ	5.31	5.00	5.51	5.76	-	5.94
4	4.0	LIJ	3.18	3.11	3.28	3.38	3.68	4.48
5	5.5	LIJ	5.91	2.58	6.14	5.91	X	4.42
6	0.5	LEJ	2.23	0.00	2.28	1.55	X	0.98
7	10.5	LIJ	8.38	2.58	8.72	8.50	X	5.44
8	9.0	REJ	7.53	4.06	7.83	7.90	-	6.18
9	3.0	LIJ	3.38	5.00	3.49	3.73	3.68	5.14
10	5.5	RIJ	5.70	6.43	5.92	6.44	9.19	6.92
11	3.5	LIJ	2.37	3.43	2.43	2.37	4.41	3.55

### Noninvasive QCU-derived CVP

**Fig. 2.**
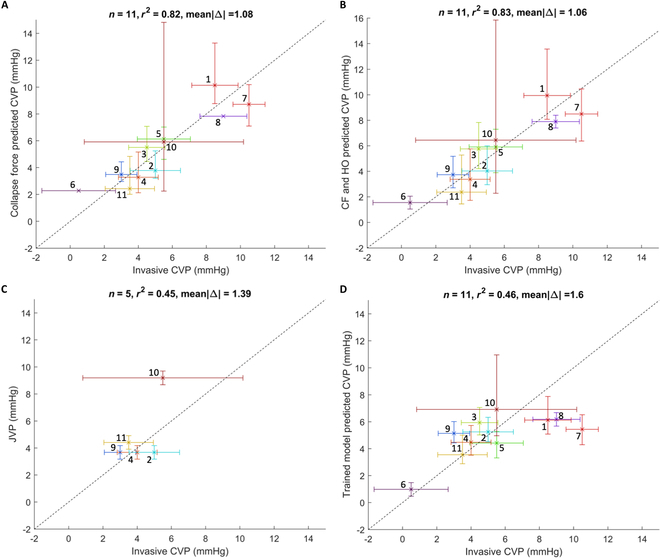
Ground truth correlation plots. Predictions versus gold standard invasive CVP measurements for 11 subjects, except for the JVP, which is satisfactorily measured on 5 subjects. Scatterplots present several measurements (vertical axis) versus the invasive CVP (horizontal axis). Error bars show quantified uncertainty in each measurement in their respective axes. The numbers nearest each measurement “x” correspond to the patient numbers in Table 2. The identity line is displayed as a black dashed diagonal to visualize error in each plot. (A) Predicted CVP based on CF measurements. (B) Predicted CVP based on CF measurements and hydrostatic pressure offset. (C) JVP in mmHg. (D) CVP predictions from the [26] linear regressor using CF measurements and hydrostatic pressure offset based on angle of inclination against average invasive CVP.

Here, we do not consider hydrostatic offset (HO). We produce the linear regression equation using CF to predict CVP.

$$CVP_{CF}=1.05*CF-0.053$$ (1)

where CF is collapse force in newtons. When compared with average invasive CVP, the *r*^2^ correlation coefficient is 0.82 (Fig. 2A) with a mean absolute error of 1.08 mmHg, and a standard deviation of 1.24 mmHg (Fig. S2A). The *P* value is 0.00013. This finding indicates a strong positive correlation of CF with CVP.

### Noninvasive QCU-derived CVP with correction for HO

We further analyze the data and account for both CF and hydrostatic pressure offset.

$$HO=10*\text{sin}\left(\theta \right)$$ (2)

$$CVP_{CF,HO}=1.05*CF+0.27*\;HO*C-0.78$$ (3)

HO is hydrostatic offset, θ is the angle of inclination of the IJV above the right atrium, and C is the conversion constant between pressure units from cmH_2_O to mmHg of 0.7356. With a hydrostatic pressure offset correction, the *r*^2^ correlation coefficient slightly increased to 0.83 (Fig. 2B) with a mean absolute error of 1.06 mmHg and a standard deviation of the error of 1.19 mmHg (Fig. S2B). The *P* value for CF is 0.00024, while that for HO is 0.44. This finding indicates a strong positive and a minuscule correlation of CF and HO, respectively, with CVP.

We also employ a quadratic regression for invasive CVP with CF and HO, producing an increased *r*^2^ correlation coefficient of 0.88 and mean absolute error of 0.81 mmHg. This result, along with its risk of overfitting, is further described in Fig. S3.

### JVP measurements

JVP was measured visually in a subset of 8 patients. For 3 of these patients, the top of the jugular pulsations could not be seen at the patients’ angle of inclination because it was either too low or too high. In this case, we determine the linear regression for the remaining 5 measurements.

$$JVP=h*\text{sin}\left(\theta \right)*C$$ (4)

$$CVP_{JVP}=1.52*\;JVP-1.59$$ (5)

We observe in Fig. 2C a squared correlation coefficient of 0.45, a mean absolute error of 1.39 mmHg, and a standard deviation of the error of 1.88 mmHg (Fig. S2C). The *P* value is 0.14. A subtler observation to point out is the range of the JVP measurement in terms of invasive CVP measurement, which is 3 mmHg. The range of the CF-based metrics in terms of invasive CVP measurement is 10 mmHg. This finding indicates a weak positive correlation of JVP with CVP with a restricted observation range.

### Trained model CVP estimation

We employ the linear regressor developed in [26] to obtain CVP estimations from the CF and the measured HO. We apply the following formula to yield a CVP estimate:

$$CVP_{\text{training}\;set}=0.42*\;CF+0.054+HO$$ (6)

A squared correlation coefficient of 0.46 is obtained when comparing these CVP predictions to the mean invasive counterpart along with a mean absolute difference of 1.60 mmHg (Fig. 2D) and a standard deviation of the error of 2.17 mmHg (Fig. S2D). The *P* value is 0.022. We note that the model tends to underestimate higher CVP values while accurately or slightly overestimating lower CVP values. We also note that this model is the only QCU-based model not generated via regression from invasive CVP measurements. Therefore, the results are purely test results as opposed to training results. Furthermore, the maximum CVP estimated by the model is 6.92 mmHg, which is attributed to the subject with the greatest hydrostatic pressure. This finding indicates a weak positive correlation of the trained model with CVP.

Conversely, we validated the CF only equation on the data from [26], finding a mean absolute error on the dataset of most similar HO of 1.07 mmHg (Fig. S4).

### Uncertainty overlap accuracy assessment

**Fig. 3.**
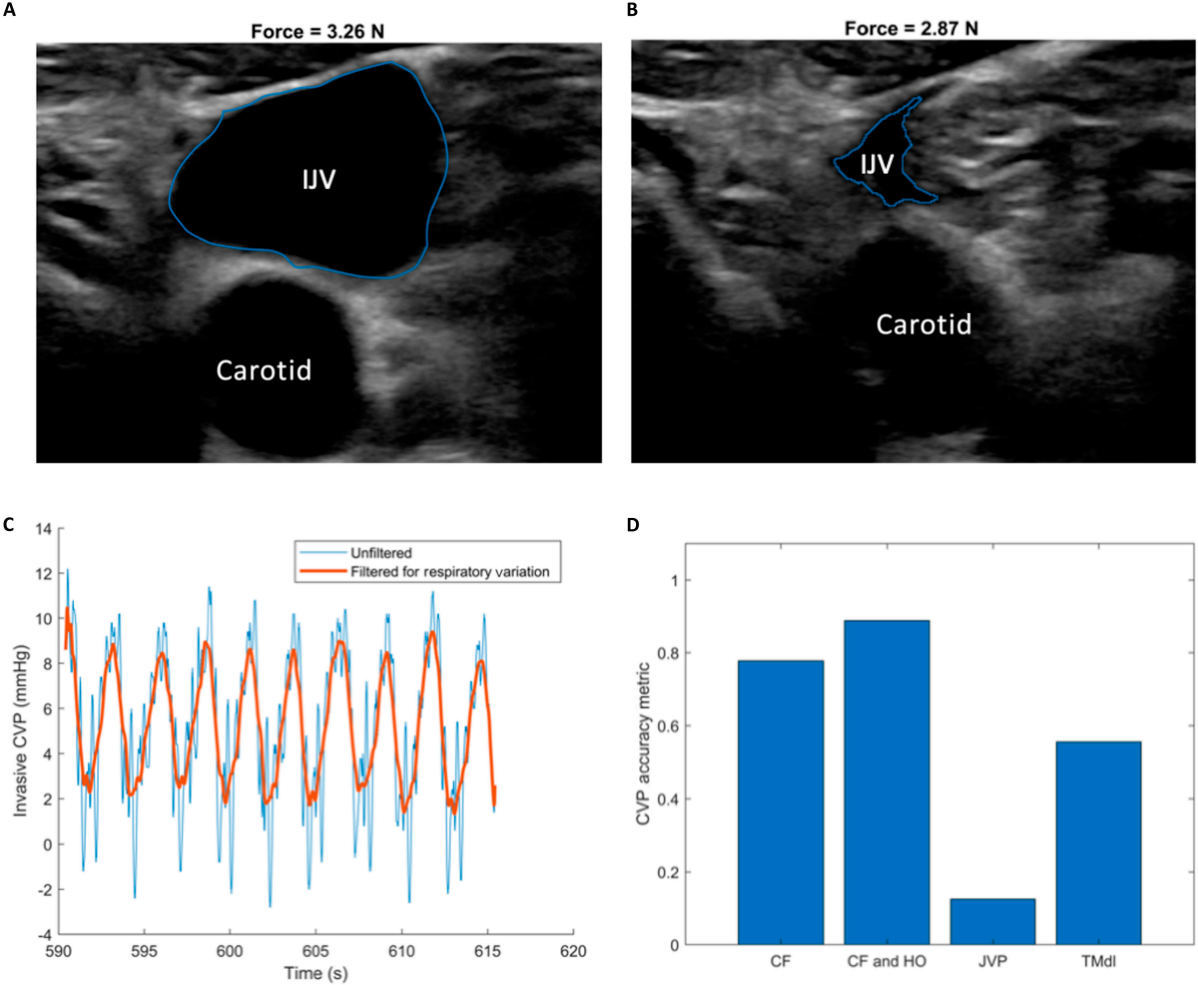
QCU uncertainty utilization. (A) Subject 10 segmented QCU image of the IJV while exhaling. (B) Subject 10 segmented QCU image of the IJV while inhaling. (C) Subject 10 unfiltered (blue) and filtered (orange) CVP waveform to highlight respiratory variation for about 25 s. (D) Bar graph showing uncertainty overlap of predicted CVP including quantified uncertainty with gold standard average invasive CVP for the population of subjects. The standard of overlap includes QCU method uncertainty and does not include invasive CVP uncertainty. CF signifies the model that uses CF. CF and HO signifies the model that uses CF and HO. JVP signifies the model that uses jugular venous pulsation height. TMdl signifies the application of the trained model from the study in [26].

The invasive CVP waveforms are observed in Fig. S1, where we can see that subject 10, experiencing hyperpnea and tachypnea, has the most respiratory variation. Subject 10 also has the most CF uncertainty in Fig. 2A to D. In Fig. 3A and B, we utilize QCU images to observe vast respiratory cycle variation in cross-sectional area. We note that the amount of force applied in Fig. 3A, with a large IJV cross-sectional area, is slightly larger than that applied in Fig. 3B, with a small IJV cross-sectional area. In Fig. 3C, we filter out the higher frequency variation of the cardiac cycle in invasive CVP to show the period and amplitude of the respiratory cycle variation over approximately 25 s.

From the above observations, the slow respiratory cycle variation is what is largely conserved in the IJV area and the cardiac cycle variation attenuates in the IJV area when moving from the right atrium to the IJV. Thus, the respiratory cycle variation is what is used from the invasive CVP waveforms to quantify uncertainty, which accounts for the horizontal error bars in Fig. 2A to D. Given that JVP is measured by noticing the highest visible pulsations of the IJV, no waveform information and only measurement uncertainty are worked into the vertical error bars in Fig. 2C. Invasive CVP uncertainty and CF uncertainty are compared in Fig. S5.

In Fig. 3D, we directly compare the standard noninvasive JVP with our novel noninvasive QCU-based method regarding their performances in predicting the gold standard invasive CVP. In terms of the QCU-derived CVP estimates, we exclude cases where the EJV is compressed due to a thrombosed IJV. When considering the JVP estimates, we excluded the 3 cases where estimation did not occur and include the 3 cases with unsuccessful estimation due to unfavorable angles of inclination as missed overlaps. Taking into account uncertainty quantification for each of our methods, we assess the accuracy of each method in comparison to invasive CVP measurement. This is done without taking the invasive CVP uncertainty into account. For each method, we analyze the vertical error bars and estimates together to determine if there is overlap with the average invasive CVP measurement. The fraction of those estimates whose uncertainty overlaps with average invasive CVP is described as each metric’s accuracy. CF alone, CF and HO, JVP, and the trained model have accuracies of 0.778, 0.889, 0.125, and 0.556, respectively. By our provided definition of accuracy, these findings indicate that the QCU-based methods are between 40% and 80% more accurate than JVP in estimating CVP.

### CVP waveform estimation

**Fig. 4.**
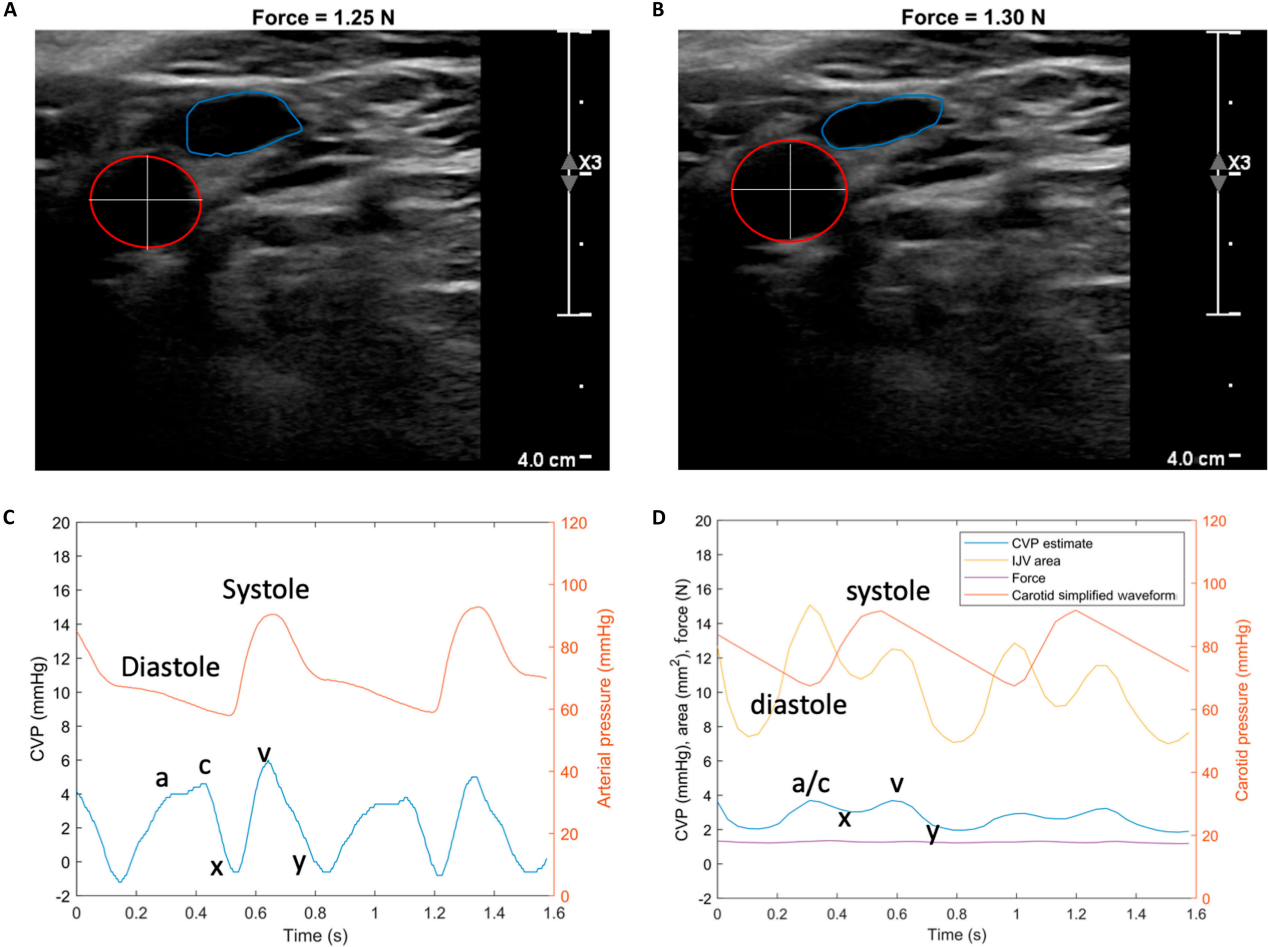
Inverse finite element model results. (A) Segmented QCU ultrasound image of the patient’s carotid artery (red) and internal jugular vein (IJV) (blue) at point “a/c” in the cardiac cycle within diastole. A white cross-hair is drawn in the carotid. (B) Segmented QCU ultrasound image of the patient’s carotid artery (red) and IJV (blue) at point “v” in the cardiac cycle within systole. An equally sized white cross-hair is drawn in the carotid. (C) Central venous pressure (CVP) measurement from an indwelling catheter in the patient’s right atrium from the MGH study (blue). A radial arterial line from the same patient is shown (orange). The components of the CVP waveform are labeled. “a” is the right atrium pumping blood into the right ventricle, “c” is the tricuspid valve closing at systolic onset, “x” is the relaxation of the right atrium, “v” is the filling of the right atrium, and “y” is the passive emptying of the right atrium once the tricuspid valve opens. (D) Inverse finite element model output of a jugular venous pressure wave (blue) with waveform components labeled, IJV area (yellow), a simplified carotid pressure waveform (orange), and force (purple).

As demonstrated in [24,26], a segmented QCU image stack of the carotid artery and the IJV is automatically attainable with our current techniques. Additionally, the pulsation of the carotid artery occurs close enough to the IJV and provides enough compression force to partially compress the IJV. In addition to attaining CF with a force ramp, we also observe the IJV when compressing with a small, relatively constant force, as indicated in Fig. 1B. By looking at the QCU images of the segmented IJV and carotid at certain points in the cardiac cycle, one can estimate the effect of the carotid pulsation on the IJV. In Fig. 4A, the IJV is relatively large and the carotid is relatively small, while in Fig. 4B, the IJV is relatively small and the carotid is relatively large. The difference in applied force between the 2 segmented QCU images is negligible.

The cardiac cycle with respect to the carotid and the IJV can be utilized to understand the cross-sectional area differences in the IJV and carotid in the 2 frames. The cardiac cycle in Fig. 4A is in diastole. Note that, at point “a/c”, a joint peak in the CVP waveform is present and corresponds to right atrial contraction followed by tricuspid valve closure secondary to isovolumetric contraction of the right ventricle. While the cardiac cycle is in systole with respect to the carotid in Fig. 4B, we observe that it is also at point “v” with respect to the IJV—a different peak in the CVP waveform, corresponding to completion of atrial filling.

Next, we examine the MGH CICU waveform data of one of the patients in the study—the invasive CVP waveform, analogous to the IJV area waveform from the QCU waveform, and the invasive radial artery pressure waveform, analogous to the carotid area waveform from the QCU segmentation. We see in Fig. 4C that the “v” peak is at a higher pressure than the “a/c” peak. We also observe that the “a/c” peak in the right atrium occurs during diastole in the radial artery pressure waveform and the “v” peak occurs during systole.

We combine the concepts from the finite element models in [24,26] to produce a 2-dimensional inverse finite element model of compression of the IJV and carotid cross-sections, observed in Movie S2, to mimic the pumping carotid influence on the IJV in close proximity to it. A forward finite element model run is displayed in Fig. S6. The inputs to the inverse model are the IJV and carotid segmentation data during the relatively constant force portion of the data capture and the average CVP estimate based on CF measurement. Figure 4D shows an IJV cross-sectional area waveform derived from the IJV segmentation and a carotid pressure waveform derived from the carotid segmentation and simplified to highlight end-diastole and peak-systole. The “a/c” peak is higher than the “v” peak in both cardiac cycles for the IJV area waveform. Unlike in [24], carotid pressure is not estimated in this study. Therefore, the radial arterial pressure waveform is used to determine the absolute value of the pressure in the simplified carotid pressure waveform. We note that Fig. 4C and D captures similar but not time-synchronized cardiac cycles.

The initial conditions for the inverse finite element model were set using the average CVP estimate based on the CF measurement for the patient. In running the model, the model converges for a given QCU frame once the IJV area measured in the forward model output is about equal to the IJV area measured in the QCU frame. To note, the model converges with CVP estimates for each frame. The output can be observed in the blue waveform in Fig. 4D. The first observation to consider in the CVP estimate waveform is that the “v” peak is slightly higher than the “a/c” peak. This differs from those of the IJV area waveform. Second, the a-wave and c-wave morphologies, evident in the invasive CVP waveform, are less distinct. Finally, the amplitude of the CVP estimate waveform is smaller than that of the invasive CVP waveform, while the mean values of each waveform are similar. These findings indicate that a simplified, dampened version of the CVP waveform can be approximated via QCU of the IJV and inverse finite element modeling.

## Discussion

Our review of the literature found that this investigation is the first study using anything similar to QCU to measure CVP in blood vessels of patients with cardiovascular disease and compare the findings to gold standard CVP. Our previous studies did not have a true gold standard comparison [25,26]. Furthermore, certain aspects were identified during the conduction of this study, which were not appreciated in previous work. These aspects included diversity of anatomical features, wide variation in hemodynamic status, and CICU constraints in time and orthostatic positioning. Although we encountered real-world adversity, we were able to obtain meaningfully positive results. Our most promising finding thus far is the strong correlation between CF and invasive CVP, even when considering that the HO differs among patients. This correlation demonstrates the potential value and use of noninvasive QCU for CVP measurement.

Drawing an analogy to blood pressure cuffs, which provide a noninvasive measure of arterial pressure through symmetric compression of the brachial artery, QCU offers a promising alternative for noninvasive CVP assessment through asymmetric compression of the IJV. Given the relatively rapid speed of data collection in our procedure (approximately 1 min), this technology has the clinical potential for frequent use and bedside monitoring of venous pressure without the need for invasive procedures.

The fact that ignoring HO did not affect accuracy of the measurement is notable. While its empirical impact was statistically nonsignificant, it is important to consider that hydrostatic pressure is theoretically expected to play a role when patient positioning deviates from a fully supine position. We believe that this result can be attributed to the limited variability in HO among the patients in our study, as all but 2 were positioned in a consistent semi-supine posture during data collection. In contrast, CF demonstrated higher variability due to patient-specific differences in vein pressure at the site of the measurement.

Furthermore, prior research in healthy individuals demonstrated that relative hydrostatic pressure changes within patients behaved as expected [24]. Additional investigation with larger datasets may provide further insights into the interplay between patient positioning, CF, hydrostatic pressure, and the accuracy of CVP estimation. Future studies could enhance the measurement of hydrostatic pressure by incorporating both distance and angle, rather than assuming a fixed 10-cm distance from the middle of the right atrium to the IJV measurement site. For the present study, however, our findings suggest that relying on CF alone is a reasonable and practical approach for predicting average CVP in clinical settings, implying the potential to improve clinical outcomes.

The quantification of uncertainty in invasive CVP with respect to respiratory variation shows a close correlation with the uncertainty in CF. The CF uncertainty considers both respiratory variation and cardiac cycle variation in the waveform of the IJV area. This suggests that while the waveform morphology of the IJV area reflects the full cardiac cycle variation from the right atrium, the amplitude of this variation seems to be significantly attenuated. In contrast, the amplitude of respiratory variation appears to be largely preserved. To gain further insights, additional data collection from patients with high respiratory variability in invasive CVP and more detailed analysis of the IJV area waveform would provide valuable information on this aspect.

When comparing CF and JVP side by side, the CF estimate is more accurate and provides more information. The CF, through QCU, not only better predicts mean invasive CVP than does the JVP (in terms of *r*^2^ and *P* values) but also gives insight into the amplitude of the venous pressure waveform. Observation of the expansion and contraction of the IJV occurs with QCU, yielding a range of observable pressure and wide error bars. Visual JVP can only pick out the peak height of the IJV pulsations, yielding only a single observable pressure and narrow error bars. Inferior vena cava diameter measurement via B-mode ultrasound is another noninvasive technique used to estimate CVP but is found to have a weaker correlation than the QCU-based methods in this study with the anatomical and respiratory cycle positions of maximum correlation (supra-iliac, end-inspiration) to have an *r*^2^ of 0.45 [13]. Furthermore, the ratio of early mitral inflow velocity by mitral annular early diastolic velocity (*E*/*e*′) is for estimating left-ventricular filling pressure and has good classification ability but tends to be less accurate in cases of decompensated heart failure [27]. The fact that the method proposed in this study demonstrates superior performance compared to the current noninvasive CVP approaches positions CF as a promising noninvasive candidate to improve standard of care.

Beyond enhancing diagnostic capabilities, QCU has the potential to streamline clinical workflows. Unlike invasive catheterization, which requires sterile conditions, ample setup time, and high expertise, QCU can be performed at the bedside or in the field in less than 1 min with modest expertise. The technique is also less dependent on patient positioning, unlike JVP, which requires a properly aligned semi-upright posture. An advantage of QCU is its potential applicability in resource-limited settings, where traditional invasive CVP monitoring is not feasible. Current invasive approaches require sterile conditions, specialized clinical expertise, and costly equipment. In contrast, QCU could be implemented using portable, battery-powered ultrasound devices with force couplings. Such devices could enable rapid, on-site CVP monitoring in mobile health units and remote patient monitoring programs.

The CVP waveform itself is useful to observe various pathophysiological conditions, one of which is tricuspid regurgitation. Here, the reflux of blood into the atrium during ventricular systole increases right atrial pressure, causing the “v” peak to arrive earlier in the cardiac cycle and contain increased amplitude and wider breadth [28,29]. Having the opposite effect, tricuspid stenosis should present with a diminished “v” wave compared to the “a” wave [30]. Finally, although cardiac tamponade should end up getting diagnosed via other means, it should alter the CVP waveform by lessening the “y” descent [31]. This added level of detail could support more informed clinical decision-making, particularly in guiding fluid management and valve replacement. Reflecting on our waveform reconstruction in Fig. 4, the increased “v” peak is present to a mild degree compared to its presence in the invasive CVP waveform.

The inverse finite element model estimate of the CVP waveform has notable similarities and differences to its invasive counterpart. The “v” peak having slightly higher amplitude than the “a/c” peak reflects the higher amplitude seen in the invasive CVP waveform, especially considering the significantly higher “a/c” peak compared to the “v” peak in the IJV area waveform. This observation in our CVP estimate occurs because the inverse finite element model accounts for the systolic pulsation of the carotid compressing the IJV when the “v” peak occurs. The mean CVP in our estimate is similar to that of the invasive waveform, but that is to be expected given that we used the average CVP estimate from CF to constrain the inverse model and the observed.

The 2 major differences between the CVP estimate and the invasive ground truth are the amplitude and the morphology. The ground truth has a higher amplitude than the CVP estimate. This could be due to inaccuracies of the model but could also be due to attenuation of the cardiac cycle variation as the pressure waveform transfers from the right atrium to the IJV, which is evidenced by lower cardiac cycle IJV area variation than respiratory cycle IJV area variation in Fig. 3. We assume that the differences in waveform morphology are not due to lack of time synchronization, but rather due to differences in waveform capture methodology, anatomical location of waveform capture, and waveform reconstruction error. It becomes apparent that the radial artery waveform exhibits a delay in relation to the venous pressure in comparison to the carotid waveform. This discrepancy is due to the radial artery’s position further down the arterial tree than the carotid and the IJV’s position as a branch of the venous tree, while the right atrium is the stem.

There remain several limitations to this study. The first is evidenced by the poor performance of the coefficients obtained by the study in [26]. Although the previously trained model slightly outperformed the JVP, it performed significantly worse than the CF by itself. Therefore, a closed-form explicit formula that reliably predicts CVP based on CF and potentially other noninvasively quantifiable parameters is not yet realized. The second is the size of the dataset. As a pilot study, this work relies on data from a limited cohort of 11 patients. While our analysis follows statistical rigor, we recognize that larger, more diverse patient cohorts are essential to strengthen conclusions and improve generalizability. Providing demographic diversity is important, as factors such as age, gender, and underlying health conditions could influence the observed correlations. Future studies with broader and more representative patient populations will be necessary to fully understand the impact of these demographic factors and to ensure that QCU-based CVP estimation can be reliably applied across diverse clinical populations. Patient vascular status is the third limitation to the study. The right IJV, most proximal to right atrium, is ideal to test, but is most often where the patient is catheterized. Therefore, the left IJV needs to be the one tested, which could contribute to worse CVP estimation accuracy. Additionally, as shown in Fig. S7, some patient IJVs are severely thrombosed; therefore, one of the EJVs needs to be tested. Fourth, the footprint of the ultrasound probe used is quite large, which requires the device to apply ample force in order to get adequate IJV compression, causing both user and patient discomfort. Decreasing the contact area to that of a normal linear vascular ultrasound probe would allow for a smaller footprint, meaning less likelihood for coming into contact with the sternocleidomastoid or clavicle, and require less force to provide the same external pressure, which should allow the patient to be more comfortable and the user to more easily compress the IJV. Finally, given the custom nature of the force coupling, a new force coupling must be designed for each new ultrasound probe to become a QCU probe for the first time. That said, the force coupling is always detachable, so it would not impede the functionality of an ultrasound probe.

QCU for noninvasive CVP estimation has significant potential for future clinical translation. The immediate next step should be a clinical trial with multiple users to compare the utility of QCU for CVP assessment in comparison to bedside JVP estimation. The central goal of this study would be to produce an explicit formula for CVP based on CF and other quantifiable factors (i.e., HO) and then to compare its accuracy to the that of the already explicit formulation of JVP. In parallel, a longitudinal study with inpatients to establish a patient-specific calibrated method with invasive CVP would be a technically easier to attain goal but would require more commitment from study participants. Furthermore, the combination of arterial and venous pressure attainment by means of simultaneous QCU imaging of the carotid artery and the IJV, given good enough arterial pressure estimation, would highly incentivize CVP measurement in a much larger patient population as arterial pressure is already an established vital sign. Such a technique could benefit cardiovascular disease outcomes by allowing heart failure to be detected and addressed earlier in disease progression.

## Materials and Methods

### Experimental and technical design

The ultrasound probe used in the study is the Philips XL14-3 xMATRIX probe, which has 3-dimensional imaging capabilities. The ultrasound system used is the Philips Ultrasound EPIQ 7C system (Philips Inc., Amsterdam, Netherlands). The load cell used to measure force is the LSB205, and force is amplified with the IAA100 differential amplifier (FUTEK Advanced Sensor Technology Inc., Irvine, CA, USA). The accelerometer used is the ADXL335 (Analog Devices Inc., Wilmington, MA, USA). Analog-to-digital conversion of the force and accelerometer data is done by the National Instruments data acquisition (NIDAQ USB6001) system, and force waveform calculation is done with a LabVIEW virtual instrument (National Instruments, Austin, TX, USA). Like previous studies, quick compressions at the beginning and end of data acquisition are used to synchronize force data and ultrasound images. The hardware setup is similar to that of the study referenced in [26]. There is no synchronization between the QCU measurements and the CICU and CSICU (cardiac surgical intensive care unit) waveforms. In order to participate in the study, patients had to have a catheterization that continuously measured right atrial pressure.

Compared to previous studies [24,26], since the test subjects are catheterized CICU patients and not healthy, usually young, graduate students, there is less asked of the test subjects and more asked of the experimenters doing the testing. The patients are not asked to adjust their positioning. Rather, the inclination angles they are currently at are the ones used and are measured by the “Angle Pro” IOS app by putting the iPhone next to the patient on the bed. Furthermore, the only experimenter directly interacting with the patient and the QCU probe is a licensed cardiologist. The other experimenter is a nonphysician graduate student whose job is to monitor the compression on the live ultrasound images and confirm whether complete occlusion of the IJV occurs. The cardiologist places the probe on top of the patient’s IJV around Sellidot’s triangle, first testing to confirm the probe is in-position to externally compress the IJV. Then, recording of force and ultrasound in a slow and linear compression as described in the Results section and Fig. 1 takes place. To ensure patient safety, if the cardiologist encounters unexpected resistance to compression of the IJV, another position is tried, another relevant vein is tried, or the measurement is abandoned altogether.

### CF measurement

The CF is defined to be the minimum amount of force it takes to completely occlude a short-axis cross-section of the IJV. Complete occlusion is determined by when the measured cross-sectional area of the IJV dips below the threshold of 0.5 mm^2^. The CF methodology is explained in further detail in [26].

### Inverse finite element model

The inverse finite element model is a finite element model of the carotid and IJV compression solving a forward problem nested inside an inverse problem. The forward problem has pressures and force application as inputs and carotid and IJV cross-sectional area as outputs. The inverse problem optimizes the IJV pressure estimate such that it produces minimal cross-sectional area error with the measured IJV area in a segmented QCU image. The inverse finite element model methodology is explained in further detail in studies in [24,26].

### Subjects and study approval

All data collection for this study has taken place at MGH in its CICU and the CSICU. Our patients were enrolled with written informed consent prior to study participation under a protocol approved by the institutional review board at MGH (protocol 2021P003587).

### Uncertainty derivation

We quantified 4 different sources of uncertainty: the CF, the hydrostatic pressure, the invasive CVP measurement, and the noninvasive JVP. CF uncertainty is based on the cardiac cycle and respiration cycle variation. When measuring CF by assessing the IJV area, respiration and cardiac cycle variation modify the area, leading to uncertainty. We quantified this variation by linearly extrapolating the minimum and maximum feasible CFs based on the IJV area waveform variation when it is near collapse, obtaining an interval of possible CFs. Hydrostatic pressure uncertainty stems from imprecision of angle measurement and population variation in distance between the middle of the right atrium and Sedillot’s triangle. We estimated this uncertainty after the CF is converted to pressure units to be 0.5 mmHg.

Invasive CVP uncertainty arose again from both cardiac cycle and respiration cycle variation. To account for this source of uncertainty, we filtered out the influence of the cardiac cycle and computed the standard deviation from the mean specifically attributed to respiratory variation. For quantifying the uncertainty interval, we considered 2 standard deviations from the mean. Finally, the JVP uncertainty comes from a previous repeatability study and whole number rounding and is 0.5 mmHg in each direction [32].

### Defining CF uncertainty

Since invasive CVP is in fact a waveform and not just a scalar, the pressure of this waveform within a subject varies with time. The QCU methodology is able to acquire frame-by-frame IJV area measurements. We indirectly observed this CVP waveform pressure variation as CF uncertainty due to the observed correlation between CF and the scalar version of measured invasive CVP.

The components of CF uncertainty are cardiac cycle variation and respiratory cycle variations. We can categorize cardiac cycle and respiratory variation by calling variation with a period of less than 1 s and resemblance to the right atrial pressure waveform components cardiac cycle variation and calling variation within a period larger than 1 s and a more sinusoidal morphology respiratory variation.

### Statistical analysis

Various statistical methods are used to produce the results presented in this manuscript. The primary goal of the analysis is to establish the relationship between QCU measurements and CVP. These methods include linear and multilinear regression analyses, as well as the calculation of squared correlation coefficients (*r*^2^), *P* values, error bars based on quantified uncertainty (mentioned above), and the standard deviation of the error. To derive the regression equations, the independent variables were identified as CF, JVP, and HO, with the dependent variable being invasive CVP. Linear regression was applied to assess the relationship between CVP and individual variables (CF or JVP), while multilinear regression analyzed the combined influence of CF and HO on CVP.

The regression models were implemented using the fitlm function in MATLAB 2021b (The MathWorks Inc., Natick, MA, USA). This function automatically estimates model coefficients, computes *P* values for each independent variable, and calculates key performance metrics such as the *r*^2^ value. The *r*^2^ value quantifies the proportion of variance in the dependent variable (CVP) that can be explained by the independent variables in the model. The statistical significance of each independent variable in the regression model is assessed using *t* tests on the regression coefficients. For each coefficient, the null hypothesis (H₀) assumes that the coefficient is zero, indicating no relationship between the independent variable and CVP. We considered a *P* value less than α = 0.05 to determine the statistical significance of the variable.

In addition to regression analysis, advanced computational techniques are employed to process and analyze the imaging data. Batched and stochastic gradient descent methods were utilized for IJV and carotid segmentation, while convolutional neural networks are applied for IJV and carotid localization. Iterative inverse modeling is used to estimate a CVP waveform, further supporting the comprehensive analysis of the QCU data. Computer software utilized to perform numerical methods consists of MATLAB 2021b and COMSOL 5.6 (COMSOL Inc., Burlington, MA, USA). MATLAB was used for the entirety of QCU image analysis. COMSOL is used to run the forward finite element model, while the inverse model that calls the finite element model forward run was in MATLAB. This methodology uses the MATLAB/COMSOL software interface LiveLink (COMSOL Inc., Burlington, MA, USA).

## Data Availability

All deidentified data, code, and materials used in the analysis for this study are available in the Supplementary Materials as Data File S1.
