# Supplementary material for: Noninvasive Quantitative Compression Ultrasound Central Venous Pressure: A Clinical Pilot Study
Source: BME Front. 2025 Mar 19;6:0115. doi: 10.34133/bmef.0115 (PMC11922486; doi:10.34133/bmef.0115)
Supplement: Supplementary 1 — Supplementary Text Figs. S1 to S7 Movies S1 and S2 Data file S1 [file bmef.0115.f1.zip › cvp_ijv_icu_BMEF_suppinfo_rev1_clean.docx]

**Noninvasive quantitative compression ultrasound central venous pressure: a clinical pilot study – Supplementary Materials**

# **CICU WAVEFORMS**

The Massachusetts General Hospital (MGH) Cardiac Intensive Care Unit (CICU) captures two invasive waveforms and one noninvasive waveform of interest in this study. The two invasive waveforms are the central venous pressure (CVP) measured from the right atrium and the radial artery pressure. The noninvasive waveform is the surface ECG. The CVP waveform has been the waveform most utilized. The radial artery pressure waveform is utilized lightly in the main text. For the purposes of this study, the ECG can be used to confirm cardiac cycle position for CVP, as it is in Figure S1. The radial artery pressure waveform can be used to do the same thing, although less precisely.

When examining the CVP waveforms of Figure S1, one notes high variation within the cohort. Waveform vary in terms of high frequency (cardiac cycle) and low frequency (respiratory cycle) content, morphology in terms of which waveform components are present and how amplitude varies, and average value.


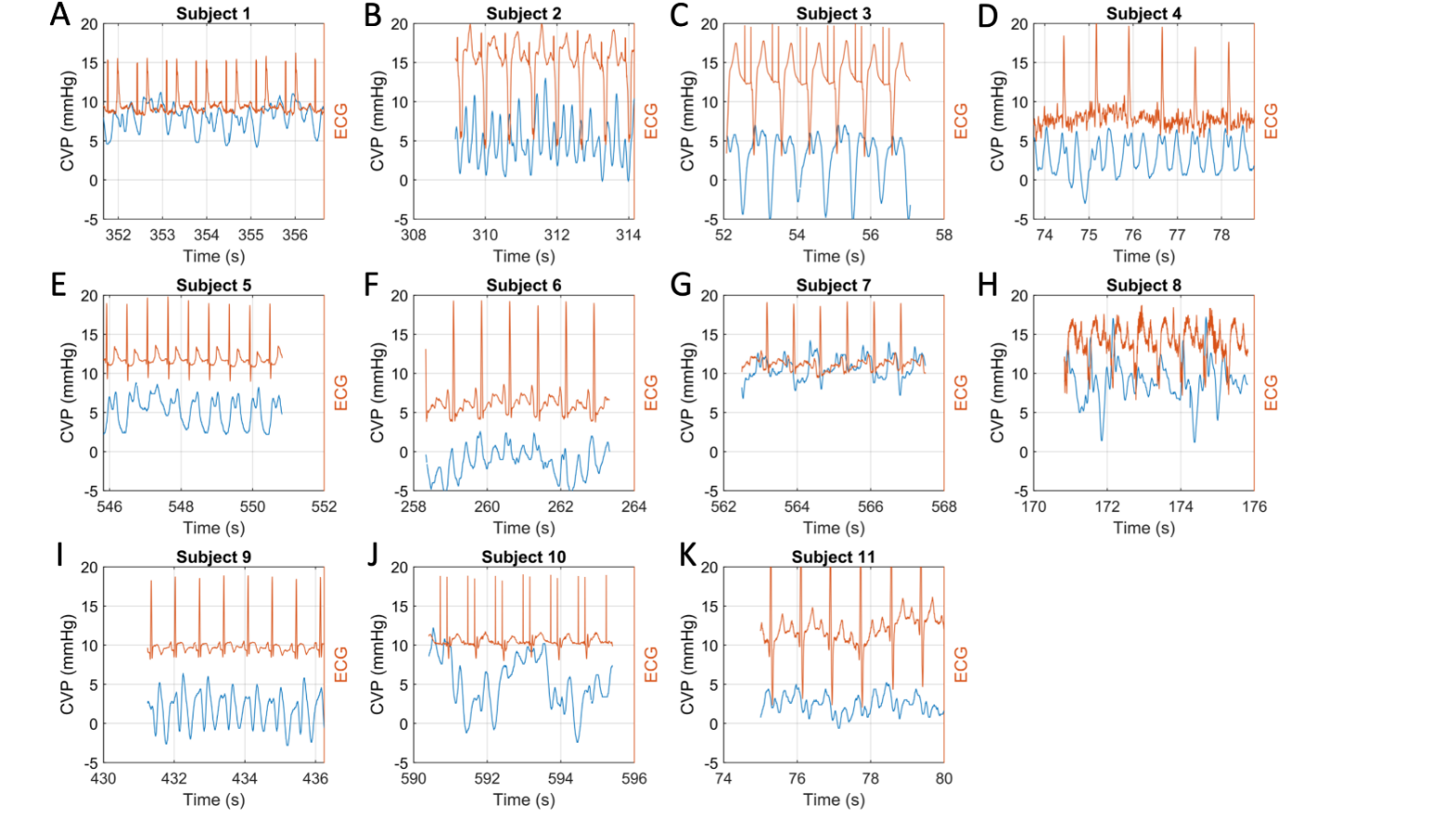


**Fig. S1. CICU Waveforms.** (A) Subject 1 invasive CVP and surface ECG waveforms. (B) Subject 2 invasive CVP and surface ECG waveforms. (C) Subject 3 invasive CVP and surface ECG waveforms. (D) Subject 4 invasive CVP and surface ECG waveforms. (E) Subject 5 invasive CVP and surface ECG waveforms. (F) Subject 6 invasive CVP and surface ECG waveforms. (G) Subject 7 invasive CVP and surface ECG waveforms. (H) Subject 8 invasive CVP and surface ECG waveforms. (I) Subject 9 invasive CVP and surface ECG waveforms. (J) Subject 10 invasive CVP and surface ECG waveforms. (K) Subject 11 invasive CVP and surface ECG waveforms.

# **CVP ESTIMATION ERROR PLOTS**

Figure S2 consists of Bland-Altman plots, also known as error plots of the average CVP estimation described in main text Figure 2. These plots are useful in seeing how error varies with the magnitude of the ground truth CVP. Measurement range, as observed by the independent variable of invasive CVP, is another thing to take away as the JVP measurement range is far smaller than the other quantitative compression ultrasound (QCU) based method because of the fixed jugular venous pulsation height inclination angles. We note that for Figure S2A-B, the mean error is zero. This is because both the collapse force predicted CVP and the collapse force and hydrostatic offset predicted CVP are regression fits.

We also remark that higher invasive CVP values yield more measurement error, which is to be expected because none of the noninvasive metrics tested here are capable of estimating CVP to be below zero. Therefore, the closer the ground truth CVP is to zero, the smaller error the estimate should have. The most extreme example of this reality is the trained model error plot in Figure S2D. The error is largely negative here because the coefficient for collapse force (0.42) in this model is significantly lower than what it was found to be in the regression (1.05). Thus, as invasive CVP increases, error gets more negative.


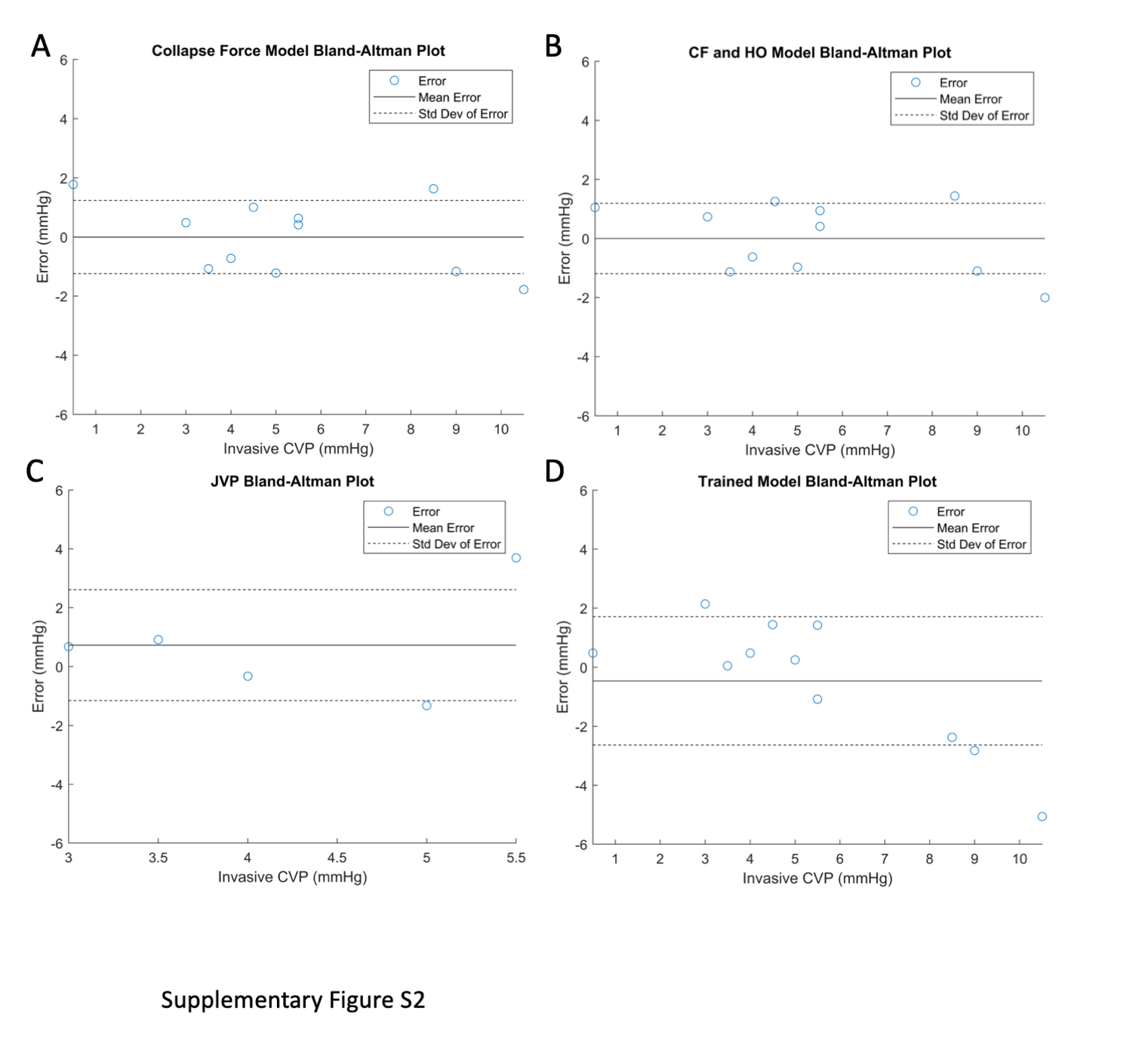


**Fig. S2. CVP Estimation Bland-Altman Error Plots.** (A) Trained model predicted CVP Bland-Altman error plot compared to invasive CVP measurements. (B) Collapse force predicted CVP Bland-Altman error plot compared to invasive CVP measurements. (C) Collapse force and hydrostatic offset predicted CVP Bland-Altman error plot compared to invasive CVP measurements. (D) JVP measurements compared to invasive CVP measurements.

# **CVP ESTIMATION WITH QUADRATIC REGRESSOR**

A quadratic regressor can be formed to estimate invasive CVP using the QCU parameter of collapse force and the hydrostatic offset parameter. Using a condensed formulation for hydrostatic offset compared to Equation 2 from the main text, $HO’$ in units of mmHg, and not explicitly showing implied multiplication, we produce the following equation:

$\mathrm{CVP}_{CF,HO}=-0.03\mathrm{CF}^{2}-0.37{\mathrm{HO}^{'}}^{2}+0.05CFHO'+1.13CF+1.76HO-1.83$ (Eq. S1)

where CF is the collapse force. The r^2^ correlation coefficient is 0.88 while the mean absolute error is 0.81 mmHg. However, the lowest p-value for any of the coefficients is 0.19, which doesn’t meet the statistically significant threshold of α = 0.05.

Figure S3A shows the correlation of the quadratic regressor with invasive CVP while Figure S3B shows the Bland-Altman error plot. While these represent the highest r^2^ value and lowest mean absolute error of the study, the high p-value, high number of fitting terms, and low number of test subjects make this result misleading due to the risk of overfitting.


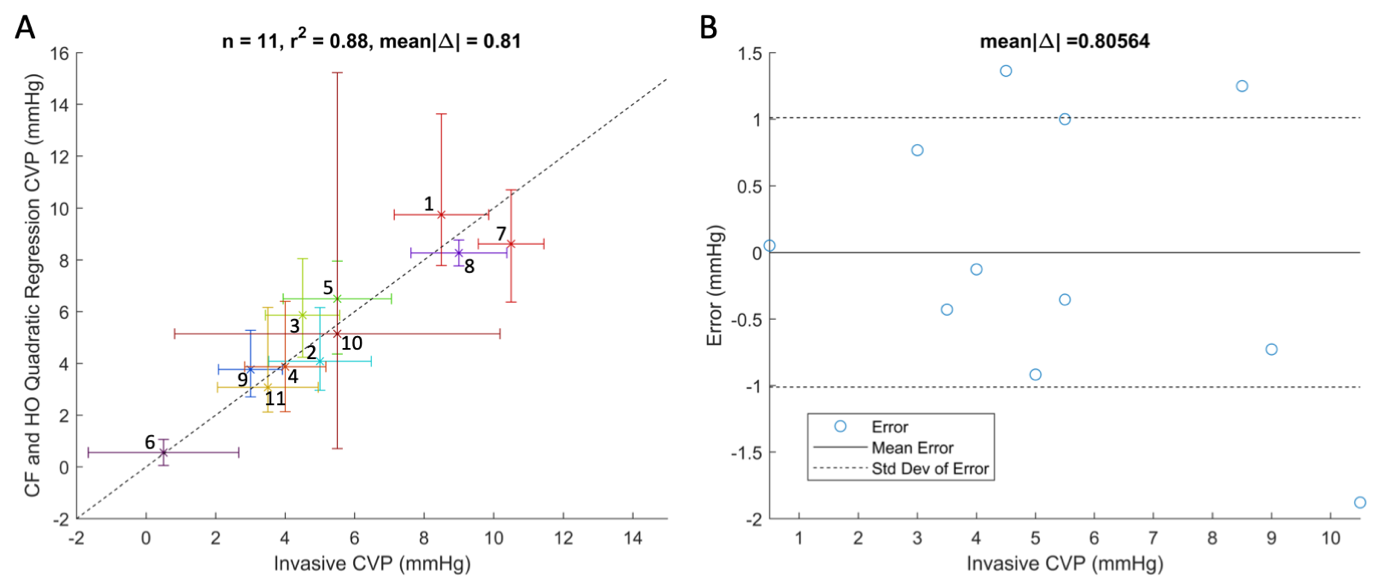


**Fig. S3. CVP Estimation with Quadratic Regressor.** (A) Quadratic regressor of CVP with collapse force and hydrostatic offset. Error bars represent the same quantities as in main text Figure 2B. Numbers refer to patient numbers in main text Table 2. (B) Quadratic regressor predicted CVP Bland-Altman error plot compared to invasive CVP measurements.

# **INDEPENDENT EXTERNAL DATASET VALIDATION**

The main text included validation of an external model from a previous study, a study of healthy volunteers on the MIT campus, in Figure 2D. Here, we look at the validation of the collapse force model generated in the study of this manuscript at MGH on two independent datasets from the MIT study. Equation 1 from the main text, with slope of about 1.05 and bias of about -0.05, is applied to the collapse force data from subjects who were completely supine and subjects who were elevated to 16 degrees above supine (around 3 cm for average height). We also note that the CVP estimates from the MIT study were produced by measuring the angle at which pulsations of the internal jugular vein (IJV) appear visible above the clavicle when slowly decreasing the angle of inclination. Thus, the MIT study did not have a true gold standard ground truth.

Figure S4A is a Bland-Altman error plot comparing Equation 1 applied to the supine data from the MIT study compared to the CVP estimates from the MIT study. We observe a large positive error with a mean absolute error of 4.29 mmHg. Figure S4B differs from panel A only in that the data used is the 16-degree elevated collapse force data, as opposed to the supine data, from the MIT study. Here, we observe a small positive error with a mean absolute error of 1.07 mmHg, similar to that same model with the MGH collapse force data.

The superior performance of the 16-degree elevated dataset is not surprising as most patients in the MGH study were elevated higher than 16-degrees above supine. Furthermore, as observed in the MIT Study (24), as CVP increases across population, the difference between the collapse force at supine and the smaller collapse force at a fixed angle above supine also increases. Hence, we should not expect such a high collapse force for a high ground truth CVP if the patient is elevated significantly above supine. These notes help explain why the first order coefficient generated for the collapse force model of this study (1.05) is substantially higher than those generated in the previous MIT study (less than 0.5 for both for supine and 16-degree elevated).

**
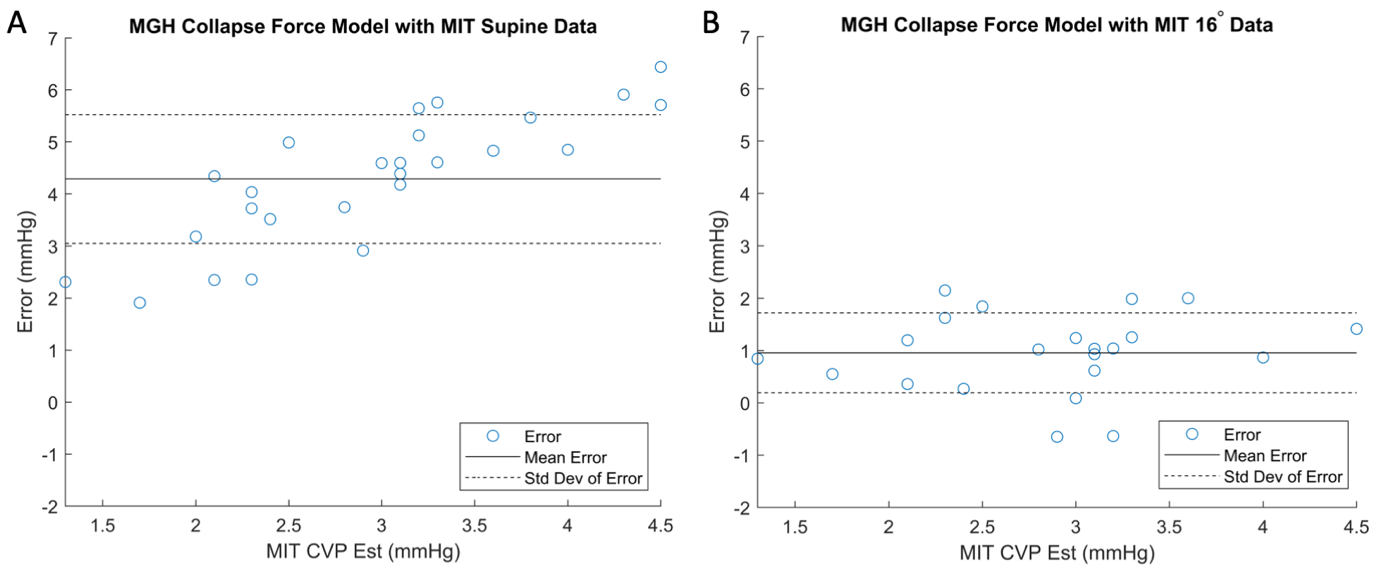
**

**Fig. S4. MGH Model Estimation of MIT Data.** (A) Bland-Altman error plot of supine collapse force data from the MIT study using the MGH collapse force model coefficients to predict the CVP estimated by the MIT study. (B) Bland-Altman error plot of 16**-**degree elevated collapse force data from the MIT study using the MGH collapse force model coefficients to predict the CVP estimated by the MIT study.

# **COLLAPSE FORCE UNCERTAINTY COMPARISON WITH INVASIVE CVP**

We now compare the uncertainty quantification from both the collapse force and the invasive CVP measurements. The two subjects with EJV compression are not included in the analysis due to low collapse force uncertainty of the EJV in comparison to the IJV. The squared correlation coefficient of both uncertainties is 0.92 (Figure S5A), while the mean absolute error is 0.52 mmHg, and the standard deviation of the error is 0.66 mmHg (Figure S5B). We also note that the highest uncertainty in invasive CVP is overestimated by the collapse force uncertainty model by over 1.5 mmHg.

Similar to the results of the uncertainty overlap accuracy of the results, the takeaway here is that the magnitude of the uncertainty in the collapse force measurement, mediated by quantitative compression ultrasound (QCU), is highly predictive of the magnitude of uncertainty in the invasive CVP measurement. What’s more, the slope of the fitted line in is greater than 1, indicating a high likelihood that the respiratory variation in CVP is fully conserved while the cardiac cycle variation is partially conserved. As we recall from the main text, only the respiratory variation is considered in invasive CVP uncertainty while the cardiac cycle variation is filtered out. Therefore, collapse force measurement, by itself, is able to give insights into not only the mean (see main text Figure 2A), but also the amplitude of the CVP waveform, independent of constant force IJV segmentation and inverse finite element modeling.


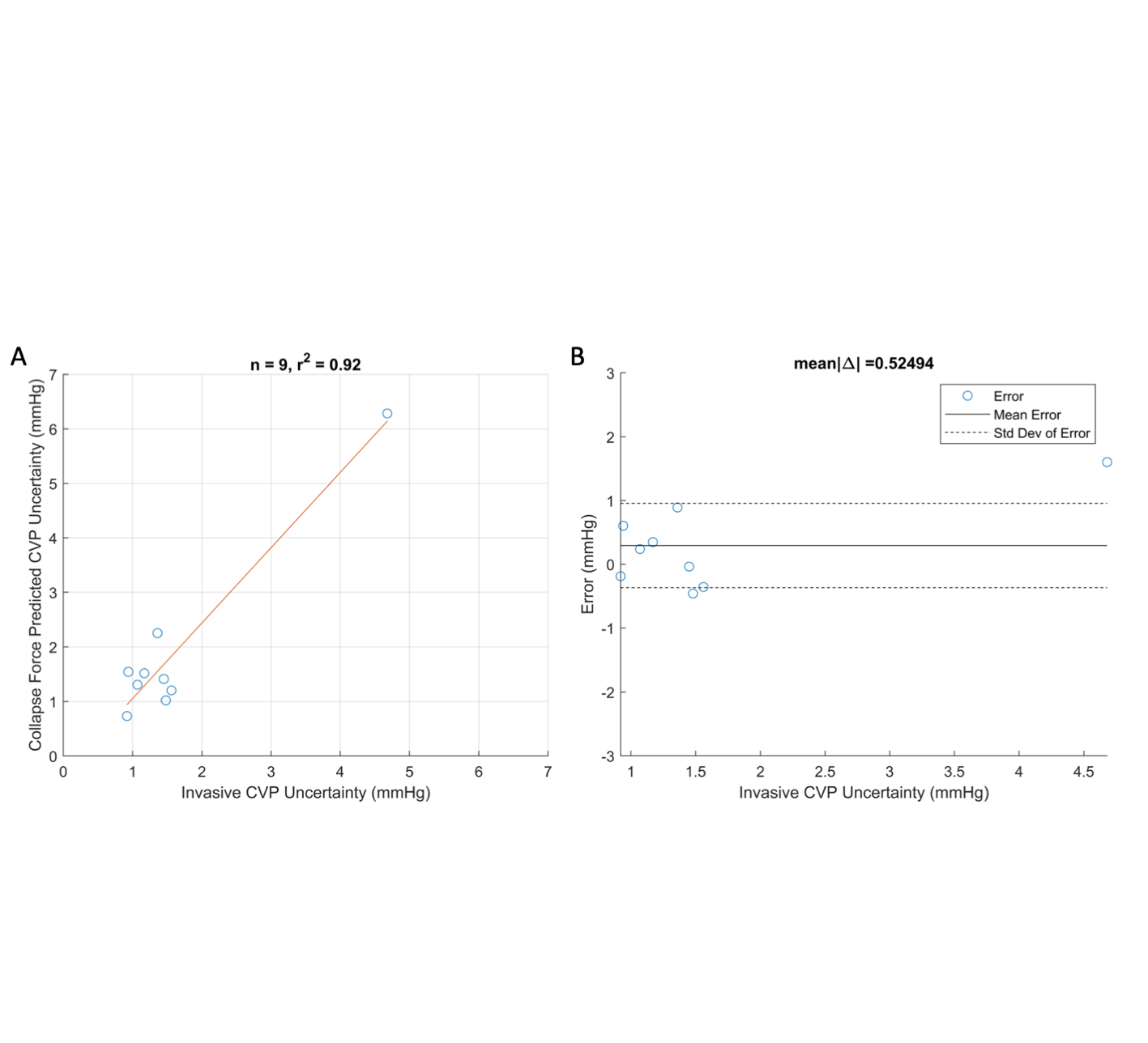


**Fig. S5. Invasive Compared to Noninvasive Uncertainty.** (A) Collapse force CVP prediction uncertainty against invasive CVP uncertainty. Line (B) Error plot of collapse force predicted CVP uncertainty compared to invasive CVP uncertainty.

# **FINITE ELEMENT MODELING**

In previous studies, COMSOL and MATLAB have been utilized with segmentation information from QCU imaging to develop an inverse finite element model to determine pressure in the carotid artery with a two-dimensional forward model *(23)* and in the internal jugular vein (IJV) with a three-dimensional forward model *(25)*. The three-dimensional model likely causes the overall model to be more physiologically accurate. However, the computational complexity is quite high and soft tissue and muscle modeling inaccuracies would remain. Therefore, a two-dimensional forward model was chosen for this study.

What’s new in this study is the modeling of the carotid artery alongside the IJV. This is crucial in terms of getting any kind of CVP waveform morphology correct due to where the carotid is in the cardiac cycle – systole or diastole. Something that was challenging for the two-dimensional model was large changes in IJV cross-sectional area output given relatively small IJV pressure input changes. To mitigate this issue, a thick, low elastic modulus shell surrounds the IJV, as can be seen in Figure S4A-B. Additionally, the carotid artery is present to compress the IJV during systole and to allow it to expand during diastole. This causes the a-wave and c-waves to be augmented and the v-wave to be diminished in the forward model IJV area waveform output. The usefulness of the carotid artery modeling is apparent in Figure S5C, where the augmented/diminished effect is successfully reproduced.


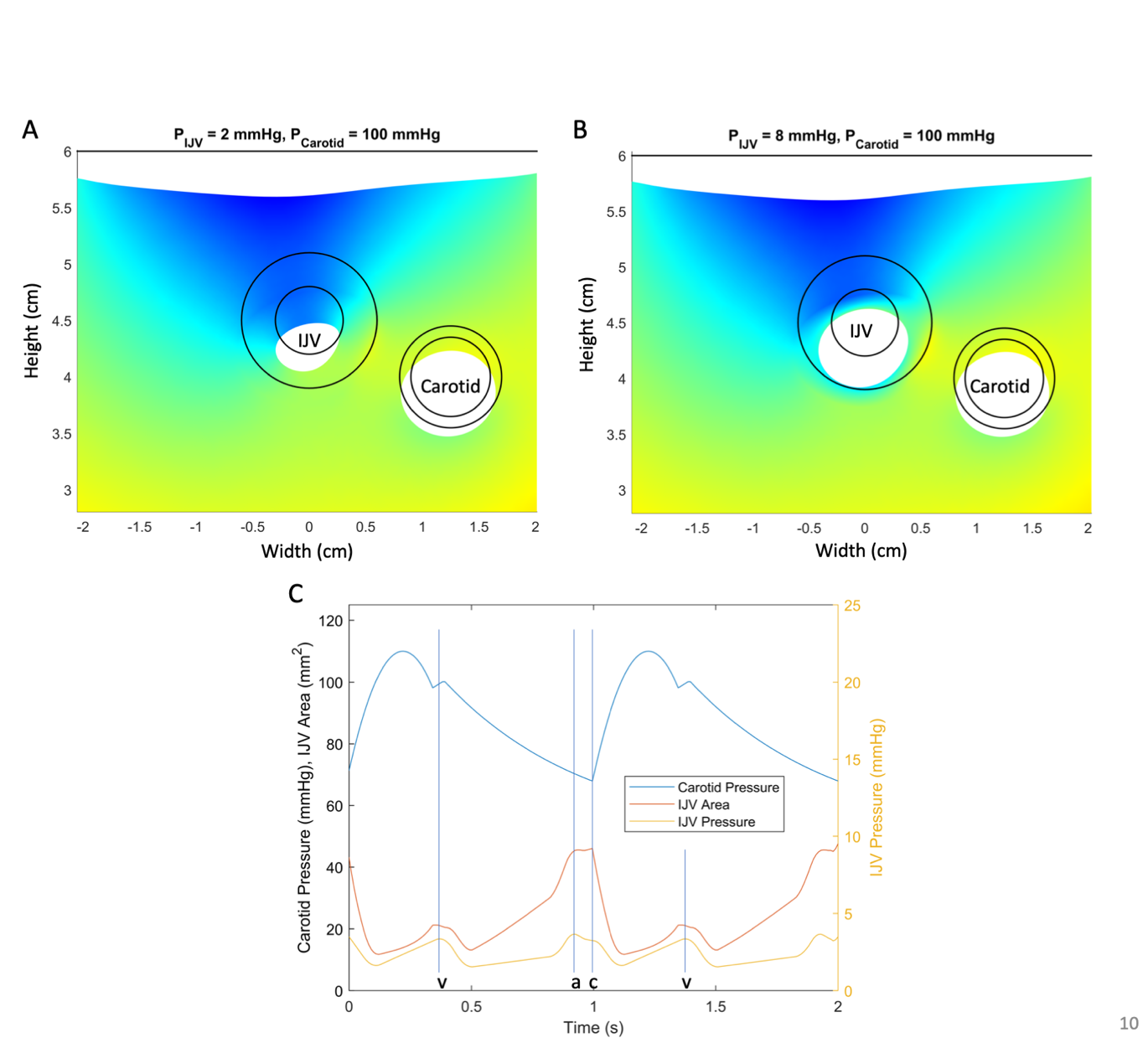


**Fig. S6. Finite Element Forward Model Runs.** (A) A forward finite element model run of the internal jugular vein (IJV) with a low elastic modulus shell and the carotid artery with a high elastic modulus shell being compressed from the top of the model. The carotid expansion alters the cross-section of the IJV. (B) A forward finite element model run with increased pressure in the IJV producing a significantly larger cross-section. (C) A forward finite element model time-domain plot of input pressures (Carotid and IJV) and output IJV area. The carotid is in systole during the v-wave and in diastole during the a-wave and c-wave.

# **STUDY LIMITATIONS FROM PATIENT VASCULAR STATUS**

Compared to previous studies, a major challenge of collecting data on CICU patients at MGH is their vascular statuses. Ideally speaking, the vein that should be used to best approximate central venous pressure that can usually be viewed and fully compressed with a QCU probe is the right internal jugular vein (IJV). However, in this study, the catheter used the measure invasive CVP is placed into the right heart via the right IJV. In addition to increased risk to the patient due to interaction with the catheter, the right IJV won’t fully occlude if the catheter is there. Thus, the left IJV must be used most often.

A further complication of patient vascular status is IJV thrombosis. Compressing this IJV, such as the one in Figure S7 would be a greater risk than doing so with the catheter due to risk of clot migration. Additionally, complete occlusion would not be possible. Subject 6 had this issue in the left IJV while subject 8 had this issue in the right IJV. In these cases, the external jugular vein (EJV) was tested instead.

#
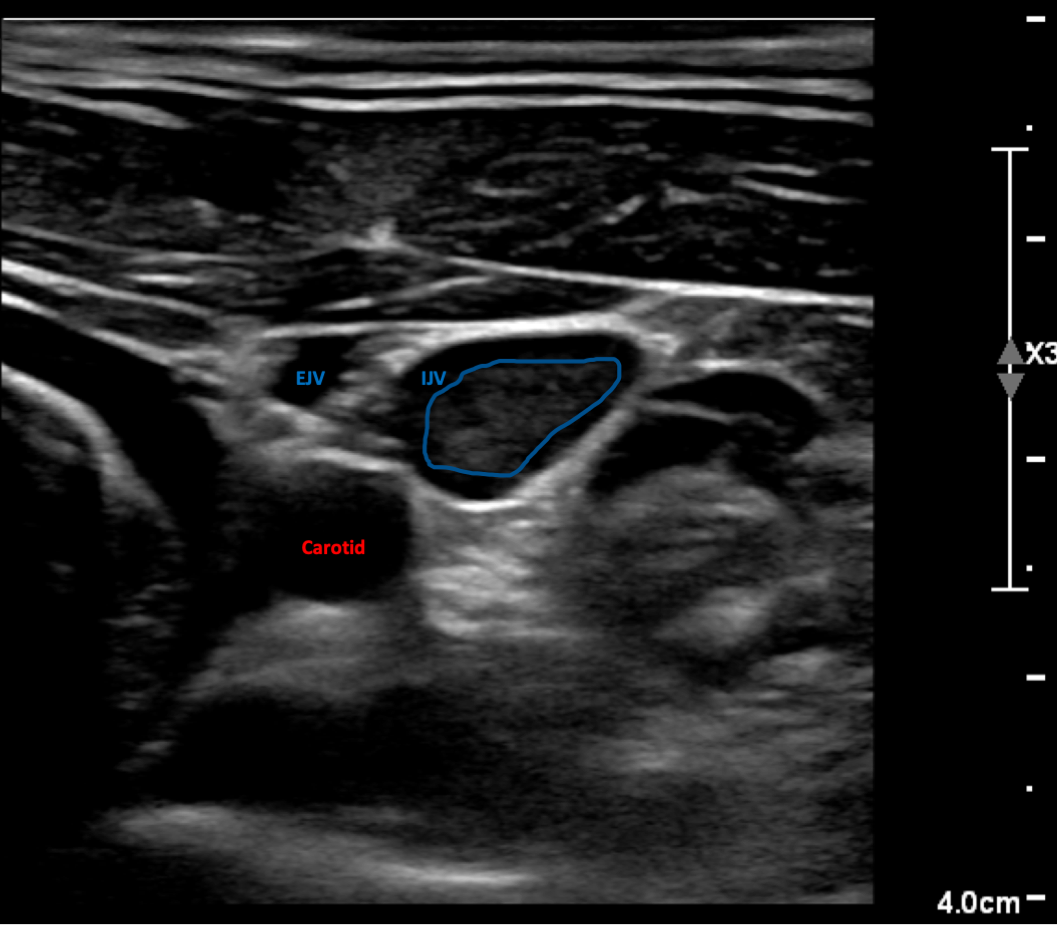


**Fig. S7. Thrombosed Internal Jugular Vein of subject 6.** This is a cross-sectional short-axis image of the left IJV EJV and carotid artery of subject 6. The blue outline attempts to trace the thrombosis cross-section in the IJV.

**NOTES ON OTHER SUPPLEMENTARY MATERIALS**

**Movie S1:** Quantitative compression ultrasound (QCU) video of entire force sweep and constant force from Figure 1. Force is in Newtons. The video is played at-speed (29 frames per second).

**Movie S2:** Segmentation video of the carotid and IJV from Figure 4. Force is in Newtons and time is in seconds. The video is slowed to about 5.5 frames per second.

**Data File S1:** All deidentified data, code, and materials used in the analysis for this study is available as a zip file as data file S1 data&code.zip. This data file consists of the deidentified ultrasound images, force measurements, and data processing results. The code necessary for data processing is also included. The data file can be accessed via the following link: <http://datadryad.org/share/6Mc9YGY5sWCQq6ND_90Z7i5YmXH-TV14DorOylzMTG4>
